# Interdisciplinary Parkinson’s Disease Deep Brain Stimulation Screening and the Relationship to Unintended Hospitalizations and Quality of Life

**DOI:** 10.1371/journal.pone.0153785

**Published:** 2016-05-09

**Authors:** Masa-aki Higuchi, Daniel Martinez-Ramirez, Hokuto Morita, Dan Topiol, Dawn Bowers, Herbert Ward, Lisa Warren, Meredith DeFranco, Julie A. Hicks, Karen W. Hegland, Michelle S. Troche, Shankar Kulkarni, Erin Hastings, Kelly D. Foote, Michael S. Okun

**Affiliations:** 1 Department of Neurology, University of Florida College of Medicine, Center for Movement Disorders and Neurorestoration, Gainesville, Florida, United States of America; 2 Department of Neurosurgery, University of Florida College of Medicine, Gainesville, Florida, United States of America; 3 Department of Psychiatry, University of Florida College of Medicine, Gainesville, Florida, United States of America; 4 Department of Clinical and Health Psychology, University of Florida College of Public Health and Health Professions, Gainesville, Florida, United States of America; 5 Department of Speech, Language, and Hearing Sciences, University of Florida College of Public Health and Health Professions, Gainesville, Florida, United States of America; 6 Rehabilitation Services, University Florida Center for Movement Disorders and Neurorestoration, Gainesville, Florida, United States of America; National Institue on Drug Abuse, UNITED STATES

## Abstract

**Objective:**

To investigate the impact of pre-operative deep brain stimulation (DBS) interdisciplinary assessments on post-operative hospitalizations and quality of life (QoL).

**Background:**

DBS has been utilized successfully in Parkinson’s disease (PD) for the treatment of tremor, rigidity, bradykinesia, off time, and motor fluctuations. Although DBS is becoming a more common management approach there are no standardized criteria for selection of DBS candidates, and sparse data exist to guide the use of interdisciplinary evaluations for DBS screening. We reviewed the outcomes of the use of an interdisciplinary model which utilized seven specialties to pre-operatively evaluate potential DBS candidates.

**Methods:**

The University of Florida (UF) INFORM database was queried for PD patients who had DBS implantations performed at UF between January 2011 and February 2013. Records were reviewed to identify unintended hospitalizations, falls, and infections. Minor and major concerns or reservations from each specialty were previously documented and quantified. Clinical outcomes were assessed through the use of the Parkinson disease quality of life questionnaire (PDQ-39), and the Unified Parkinson’s Disease Rating Score (UPDRS) Part III.

**Results:**

A total of 164 cases were evaluated for possible DBS candidacy. There were 133 subjects who were approved for DBS surgery (81%) following interdisciplinary screening. There were 28 cases (21%) who experienced an unintended hospitalization within the first 12 months following the DBS operation. The patients identified during interdisciplinary evaluation with major or minor concerns from any specialty service had more unintended hospitalizations (93%) when compared to those without concerns (7%). When the preoperative “concern” shifted from “major” to “minor” to “no concerns,” the rate of hospitalization decreased from 89% to 33% to 3%. A strong relationship was uncovered between worsened PDQ-39 at 12 months and increased hospitalization.

**Conclusions:**

Unintended hospitalizations and worsened QOL scores correlated with the number and severity of concerns raised by interdisciplinary DBS evaluations. The data suggest that detailed screenings by interdisciplinary teams may be useful for more than just patient selection. These evaluations may help to stratify risk for post-operative hospitalization and QoL outcomes.

## Introduction

Deep brain stimulation (DBS) has proven to be an effective therapeutic modality for select patients with Parkinson’s disease (PD), essential tremor (ET), dystonia, and other neuropsychiatric disorders [1]. Proper selection of surgical candidates is considered critical not only for successful DBS, but also to prevent complications by adequately planning surgical-related procedures [2–4]. DBS complications have been classified as surgery-, device-, and stimulation-related issues and complications can be a cause of a prolonged length of hospital stay or re-hospitalization after surgery. Most common surgery-related complications, which present in the acute setting, are neurovascular disorders, seizures, postoperative confusion, and infections. Hardware- or stimulation-related issues are present as subacute or longer-term effects. The reported incidence of complications vary in the published literature, with most of the surgery-related side effects presenting in less than 5% of cases, and device- and stimulation-related side effects varying from 1–75% [5].

DBS has shown to be a risk factor for unintended hospitalization in PD patients, and DBS PD patients are 2.5 times as likely to be hospitalized than non-DBS PD patients [6]. However, specific DBS complications leading to hospitalization have not been reported in large series. To date, there are no universally accepted criteria for assessing DBS candidacy, and selection has been based largely on expert consensus [7]. Many DBS centers utilize an interdisciplinary approach to determine the appropriate candidate for surgery. At our institution, we perform a multi-specialty interdisciplinary preoperative assessment followed by a team discussion of risks and benefits classifying patients in levels of surgical concern in order to decide on candidacy. This interdisciplinary assessment is widely felt to be the optimal model for the delivery of patient centered care for PD patients, however there is wide variability in practice and there are little data to support its implementation and use.

In an effort to identify patients who were at higher risk for hospitalizations post-surgery, with the ultimate goal of guiding follow up and interventions that can reduce or prevent unintended hospitalizations, we aimed to compare the history of UH following DBS with the concerns reported during the interdisciplinary DBS screening evaluation. We also compared postsurgical complications, clinical outcomes, and quality of life (QoL) measurements between groups. Additionally, we reported the issues uncovered by each DBS screening specialty team contributing to the decision on DBS surgery candidacy.

## Methods

The University of Florida (UF) Institutional Review Board (IRB) approved the study. All patients provided informed consent according in the IRB approved UF INFORM protocol. Patient records were anonymized and de-identified prior to analysis. The UF INFORM database holds data of over 9,000 patients, including information of the DBS and clinical follow-up data.

### Study design, setting, and participants

An observational cohort study was conducted at the UF Health Center for Movement Disorders and Neurorestoration. Patients with a diagnosis of PD who had DBS implantations from the period of January 2011 to February 2013 were selected for the study and evaluated at 6 and 12 months post DBS surgery. The diagnosis of PD and the decision to screen for DBS surgery was made by a movement disorder trained specialist based on current expert recommendations [8, 9]. There were 164 candidates screened by the UF interdisciplinary DBS team and 133 (81%) were approved for DBS surgery. Thirty-one patients (19%) were excluded either because they were previously implanted at another medical center or were not considered appropriate candidates for surgery by the DBS team.

As part of our Center’s standard of care, all DBS candidates were pre-operatively and independently evaluated by seven interdisciplinary team members. The team members consisted of a movement disorders trained neurologist, a functional neurosurgeon, a neuropsychologist, a psychiatrist, a physical therapist, an occupational therapist, and a speech-language pathologist. The risks and potential benefits for each DBS candidate, as well as the proposed surgical interventions, were discussed during an interdisciplinary team meeting requiring the input of all seven specialties, and consensus approval was reached prior to any intervention.

### Data sources and measurements

Demographics and clinical variables were obtained from the UF INFORM database complemented by each patient’s electronic medical record. The variables documented included gender, age at surgery, disease duration, the Unified Parkinson Disease Rating Scale (UPDRS) part III at baseline (“off” and “on” medication scores), at 6 and 12 month post DBS surgery (“off” medication “on” stimulation, and “on” medication “on” stimulation scores), and the 39-item Parkinson Disease Questionnaire (PDQ-39) was also obtained at baseline and at 6 and 12 months post DBS [10]. The PDQ-39 is a self-reported quality of life scale composed of 8 subscores (mobility, activities of daily living, emotional, stigma, social, cognition, community, and discomfort) graded in a Likert-type scale (never, occasionally, sometimes, often, and always) where higher scores represent a better QoL.

### Outcomes

The UF interdisciplinary team classified all DBS candidates as having major, minor, or no concerns for future DBS therapy based on each team’s clinical evaluations. A concern was defined as a clinical finding, which could place an individual patient at risk for post-surgical complications. These concerns were stratified as, 1. Major Concern: the risk of surgery possibly exceeded the benefit; 2. Minor Concern: a potentially increase the risk of DBS existed, but the benefits outweigh the risks; 3. No Concern: no concerns for surgery were observed (Table 1 details examples of concerns raised by services during interdisciplinary discussion). Although the evaluations were based on detailed quantitative assessment, the final discussion and concerns were expressed in qualitative manner. Assessments used by Neuropsychology and included the Depression Rating Scale (DRS), Wechsler Abbreviated Scale of Intelligence (WASI), Full Scale Intelligence Quotient (FSIQ), Working Memory Index (WMI), Processing Speed Index (PSI), the Beck Depression Inventory (BDI), and the Apathy scale (AS). Psychiatry used the Hamilton Anxiety Rating Scale (HAM-A), Hamilton Depression Rating Scale (HAM-D), Young Mania Rating Scale (YMRS), and the Yale-Brown Obsessive Compulsive Scale (YBOCS). The physical therapy team used certain criteria to determine fall risk that included clinical judgment combined with quantitative measures: previous history of falling and performance on the Berg Balance Scale, timed up and go, and the 10-meter walk test. Speech and swallow therapists used the Communicative Effectiveness Survey (CES) and a dysphagia-specific quality of life (SWAL-QOL) and quality of care (SWAL-CARE) scales. In this analysis concerns from each specialty were given equal weight regardless of the individual specialty. DBS approved candidates were informed of their risks and concerns and it was therefore possible to have one or more specialties express a major concern and for an individual patient to still receive the operation.

**Table 1:** Examples of concerns raised by specialties during discussion of DBS candidacy.

Specialty	Major Concern	Minor Concerns
Neurology	Revision of diagnosis (e.g. PD to atypical parkinsonism); lack of levodopa response or poor levodopa response	Age, comorbidities (e.g. DM, HTN); Disease characteristic that is not of primary concern to the patient but may be unresponsive to DBS; Pacemaker; Seizure disorder
Neurosurgery	***Aggregate high surgical risk*:** advanced age, HTN, DM, obesity or malnutrition, smoking/COPD, cardiac disease, anticoagulation, h/o anesthetic complications. ***Low predicted benefit*:** predicted poor response to DBS based on patient generated prioritized list of problems affecting QOL	Brain imaging findings (e.g. atrophy, prior stroke, structural lesion), anticoagulation, pacemaker, previous neurosurgery
Neuropsychology	Dementia as evidenced by impairment in more than one neuropsychological domain on formal testing. Atypical profile for Parkinson patient (e.g. prominent anomia; simultagnosia)	Mild to moderate cognitive impairment but likely able to tolerate DBS surgery
Psychiatrist	Active unstable psychiatric disease (bipolar disorder, depression, etc.); Psychosis; Active suicidal ideation; Active and untreated alcoholism or other substance abuse disorder	Identified and managed depression, anxiety, impulse control disorders, dopamine dysregulation syndrome, substance abuse disorder
Physical therapy	Fall risk; Primary motivation for DBS surgery would be resolution of gait and balance problems	Gait, freezing and balance problems however these issues are not the primary objective of the DBS surgery
Speech and swallow pathologist	Moderate to severe dysphagia and aspiration risk. Speech and swallowing improvement are major motivators for DBS surgery. Patients with moderate to severe dysphagia preoperatively are placed NPO postoperatively until the speech clinician evaluates swallowing function. Atypical speech-language profile for PD (e.g. mixed dysarthria more associated with an atypical parkinsonism such as hypokinetic-ataxic or hypokinetic-spastic, prominent anomia)	Mild to moderate dysarthria and/or dysphagia; speech problems with an expectation for benefit; desire for improved speech but this is not the primary motivation for surgery
Occupational therapy	Unable to perform many ADLS even in the best dopaminergic on state	Mild to moderate ADLS issues but with reasonable expectations for benefit

DM: Diabetes Mellitus; HTN: Hypertension; COPD: Chronic Obstructive Pulmonary Disease; ADLS: Activities of daily living; NPO: nothing by mouth (non per os); DBS: Deep Brain Stimulation; ADL

For the assessment of the primary outcome, patients were asked about the occurrence of any hospitalization during the previous year at the 12-month post surgery clinic visit. If a hospitalization was reported, this was then verified by reviewing the patient’s electronic medical record. Any hospital admission for a PD- or a non- PD related causes were considered as hospitalization. The reason of hospitalization was also documented. Hospitalizations for DBS lead implants or Implantable Pulse Generator (IPG) changes were not included. Postsurgical complications presenting within the 1^st^ year after DBS surgery were documented, including postoperative infections, systemic or device-related, falls, post-operative mental status changes, or local skin changes around the incision site, regardless of the presence or absence of hospitalization. The complications were classified as related to DBS or not related to DBS in accordance with a previously performed study [2].

### Statistical Analysis

Patient characteristics, clinical, and QOL assessments were compared between UH-positive and -negative groups using the Wilcoxon rank-sum test. Fisher exact test was used to assess UH by levels of concerns from pre-surgery assessments. The comparisons of the DBS and non-DBS related data in UH, falls, and infection were analyzed using the Exact binomial test. The number of concerns and UH were compared using the Cochran-Armitage analysis. To identify potential predictors for UH following DBS, multiple logistic regression analyses were used considering the presence of UH as a dependent variable while age, disease duration, pre-and post-operative severity of motor symptoms (on and off UPDRS), pre and post-operative PDQ-39 were used as independent variables. All calculations were performed using SAS software, version 9.1.3 (SAS Institute, Cary, NC, USA). Statistical significance was set at p < 0.05.

## Results

### Participants

A total of 164 PD candidates for DBS were assessed by the UF interdisciplinary team, where 133 (81.1%) patients were considered appropriate for DBS surgery. All completed follow-up and were included in the final analysis. The cohort was composed of 66.4% males, which had a mean age at surgery of 62.8 (SD 8.9) years, with mean disease duration of 11.4 (SD 5.1) years. Mean UPDRS part III preoperative “off” and”on” medication scores were 38.6 (SD 10.7) and 24.7 (SD 9.7), with mean postoperative “off” medication “on” stimulation score of 39.5 (SD 11.7) and “on” medication “on” stimulation score of 26.9 (SD 9.9) at 6 months and 34.5 (SD 9.5) and 25.1 (SD 9) at 12 months post DBS implantation. Mean preoperative PDQ-39 score was of 258.1 (SD 127), with a mean score of 202.9 (SD 106.4) at 6 months and of 193.5 (SD 114.3) at 12 months follow-up.

### DBS team outcomes

**Fig 1:**
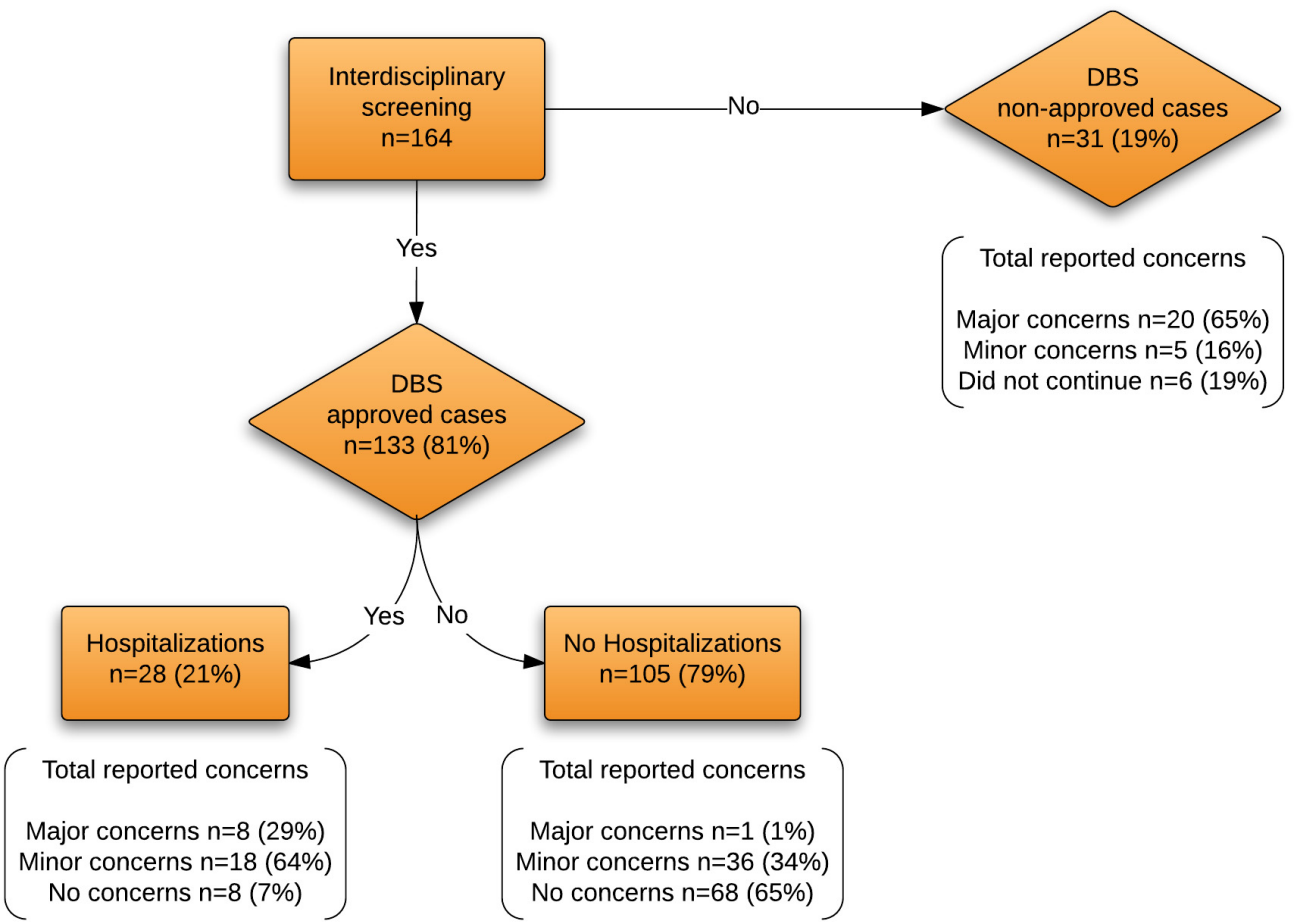
Number of patients per level of concern for those with and without UH.

Forty-seven percent (63/133) of the appropriate DBS candidates were classified as having either major or minor concerns. Nine patients were classified under the major concern group, 54 had minor concerns, while 76 patients had no concerns at all. Of the 31 cases that did not go to surgery, 20 (65%) had major concerns, 5 (16%) had minor concerns, and 6 (19%) did not continue with the evaluation. Participants at each stage are shown in Fig 1.

### Characteristics of hospitalized versus non-hospitalized patients

A total of 28 (21%) reported cases of UH were documented from the 133 patients who underwent DBS. Four of these patients had repeat UH during the period studied. Demographic and clinical characteristics between the UH (+) and UH (-) groups are summarized in Table 2. No demographics and clinical differences were observed between groups. Although the preoperative QoL was similar between groups, patients with a history of UH had significantly lower scores at 12 months post-surgery (p = 0.002). There was a significant difference between DBS-related and DBS-unrelated UH (18% vs. 82%, p < 0.001), summarized in Table 3. The primary leading causes of UH in the total cohort were falls and infections (Table 4). No significant differences in frequency of falls and/or infections were observed between DBS-related and DBS-unrelated UH. Six out of the 31 cases (19%) who did not undergo DBS surgery had a hospitalization during the next following 12 months of the DBS interdisciplinary screening.

**Table 2:** Demographic and Clinical Characteristics of Patients (n = 133).

	Unintended Hospitalization (n = 28)	Without Unintended Hospitalization (n = 105)	P value
Male, (%)	21 (75%)	66 (64%)	0.368
Age at surgery, years (M ± SD)	64.8 ± 8.7	62.3 ± 9.0	0.239
Disease duration, years (M ± SD)	12.2 ± 5.9	11.3 ± 4.9	0.758
UPDRS part III (M ± SD)			
Pre-op “off” medication	41.7 ± 9.3	37.8 ± 10.9	0.118
Pre-op “on” medication	26.6 ± 10.6	24.2 ± 9.4	0.380
6 months post-op “off” med “on” stim	41.3 ± 10.4	39.0 ± 12.0	0.424
6 months post-op “on” med “on” stim	28.2 ± 9.1	26.5 ± 10.2	0.468
12 months post-op “off” med “on” stim	36.4 ± 6.1	34.1 ± 10.11	0.300
12 months post-op “on” med “on” stim	27.8 ± 8.0	24.5 ± 9.2	0.113
PDQ-39, (M ± SD)			
Pre-op	241.2 ± 129.1	262.7 ± 126.8	0.399
6 months post-op	228.9 ± 97.6	196.3 ± 108.1	0.133
12 months post-op	241.6 ± 110.6	180.8 ± 112.6	0.028
Change in PDQ-39 at 6 months, (M ± SD)	10.27 ± 63.92	-19.24 ± 36.42	0.039
Change in PDQ-39 at 12 months, (M ± SD)	28.53 ± 86.86	-25.62 ± 38.08	0.002

UPDRS: Unified Parkinson’s Disease Rating Scale; PDQ-39: Parkinson’s Disease Questionnaire.

**Table 3:** Relationship between DBS-related and DBS-unrelated hospitalizations.

	Total	DBS-related causes	DBS-unrelated causes	P value
UH	28	5 (17.9%)	23 (82.1%)	< 0.001
Fall	25	10 (40.0%)	15 (60.0%)	0.424
Infection	9	5 (55.6%)	4 (44.4%)	1.000

UH: Unintended hospitalization; DBS: Deep Brain Stimulation

**Table 4:** Primary reasons of reported unintended hospitalizations following DBS surgery.

Reason	No.
Fall	5
Pneumonia	2
Kidney infection	1
Wound infection	2
Lead infection	1
Syncope	3
Venous infarction	2
Deep venous thrombosis	1
Anxiety	2
Seizure	1
Diarrhea	1
Spinal canal stenosis	1
Intestinal blockages	1
Chronic heart failure	1
Orthostatic hypotension	1
Transient ischemic attack	1
Muscle weakness	1
Pulmonary nodule resection	1
Total	28

### Interdisciplinary Concerns

**Fig 2:**
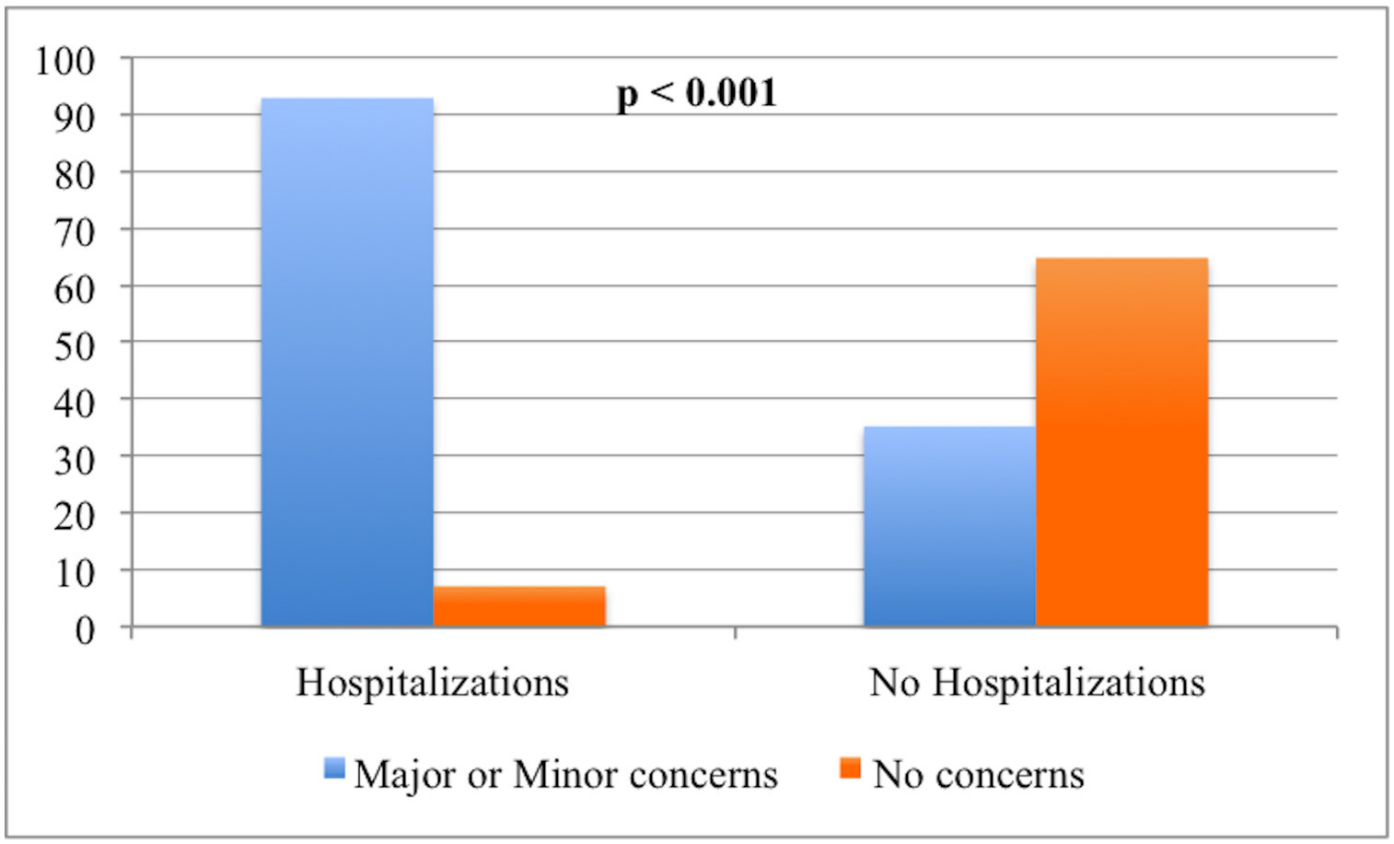
Relationship between unintended hospitalizations and level of concern.

The frequency of any major or minor concerns in the group reporting hospitalizations was significantly higher when compared to those who did not have a concern (92.9% vs. 35.2%, p < 0.001), as shown in Fig 2. The frequency of reported hospitalizations decreased from 88.9% in those with “major” concerns, to 33.3% in those with “minor” concerns, to 2.9% in patients with “no” concerns (Table 5). The higher the level of concern, the higher the rate of UH (p < 0.001). These results are summarized in Table 6.

**Table 5:** The correlation between concerns and unintended hospitalization.

	Number, n = 133	UH positive, n = 28 (%)	UH negative, n = 105 (%)	P value
Major concerns	9	8 (88.9)	1 (11.1)	N.A.
Minor concerns	54	18 (33.3)	36 (66.7)	N.A.
No concerns	70	2 (2.9)	68 (97.1)	< 0.001

UH: Unintended hospitalizations.

**Table 6:** Correlation between the number of concerns and unintended hospitalizations (P < 0.001).

Number of possible reported concerns per patient	Number of times reported	UH positive, n (%)	UH negative, n (%)
6	0	0	0
5	1	1 (100%)	0
4	2	2 (100%)	0
3	6	5 (83.3%)	1 (16.7%)
2	4	1 (25%)	3 (75%)
1	50	17 (34%)	33 (66%)
0	70	2 (2.9%)	68 (97.1%)

**Fig 3:**
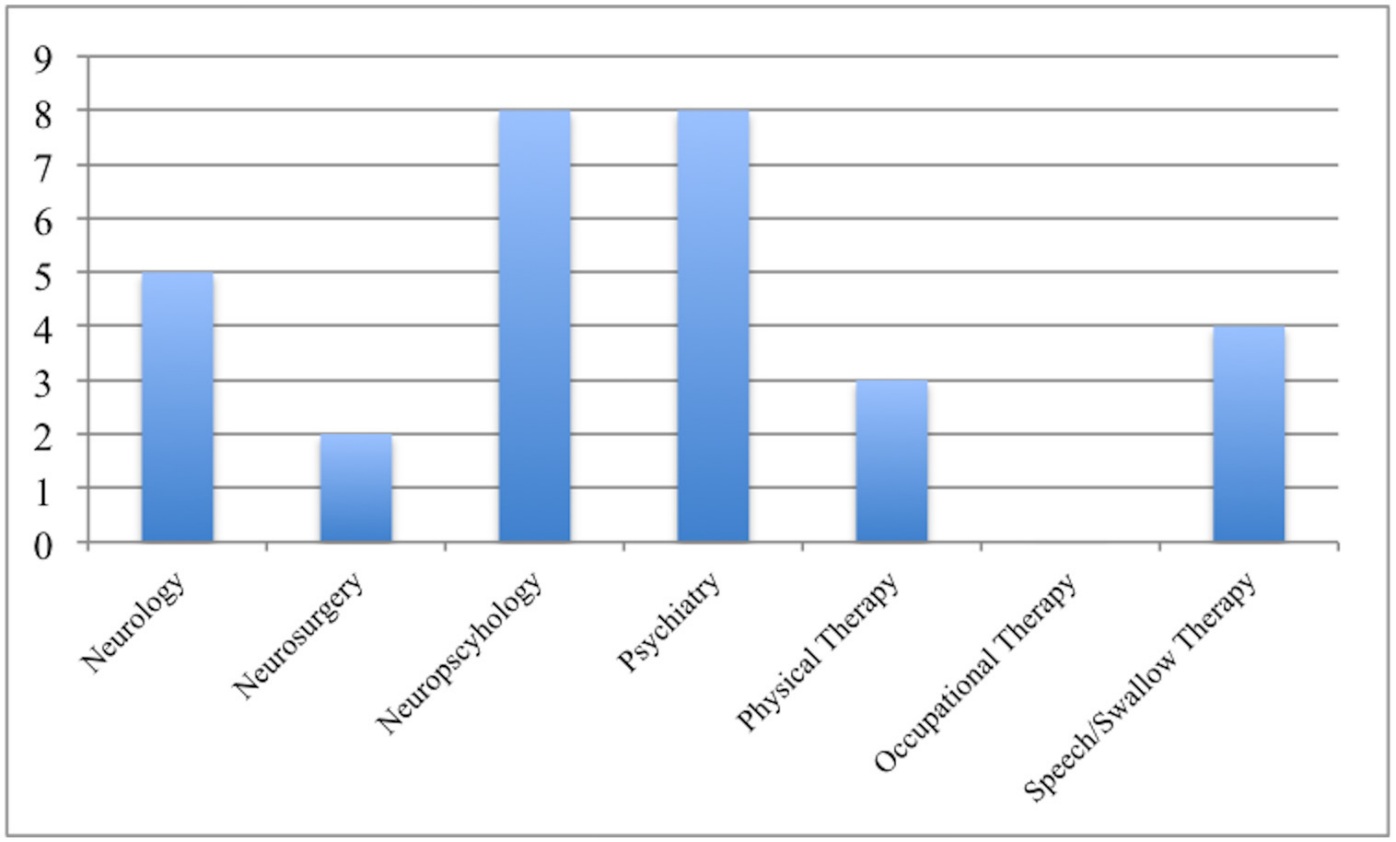
Number of concerns reported per evaluating service on those patients with UH during the interdisciplinary meeting.

**Fig 4:**
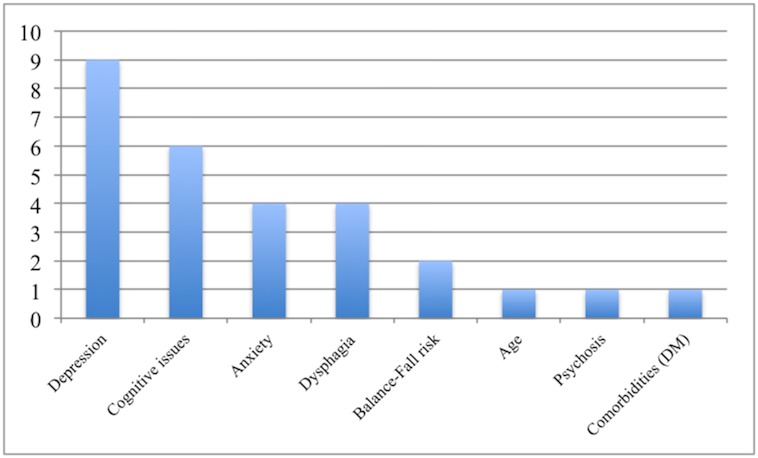
Issues cited as contributing to reservations reported by each service during screening.

Of the patients that had an UH, the three most common specialties giving rise to concerns were psychiatry, neuropsychology, and neurology (Fig 3). The leading issues cited as contributing to the team’s level of concern in the hospitalized cohort were depression, cognitive concerns, and anxiety (Fig 4).

### Post-DBS Complications

No permanent neurological sequel or death was observed after surgery in this cohort. Transient DBS-related complications included confusion in three patients, seizure in two patients, stroke in one patient, hemorrhage in one patient and deep venous thrombosis in one patient. Two patients had an IPG infection and one patient had a hardware malfunction that required both the lead and neurostimulator to be removed.

### UH Predictors

No significant clinical differences were observed between those reporting a UH with those not reporting a UH. The only significant relationship was uncovered in the postoperative PDQ-39 score at 12 months following DBS, where patients with a PDQ-39 summary index of 200 or higher were more likely to have UH (p = 0.004). The results are summarized in Table 7. The DBS implanted targets of the 24 patients who reported an UH were: 8 bilateral GPi, 5 unilateral GPi (1 with additional unilateral VIM), 5 bilateral STN, 3 unilateral STN, 1 unilateral STN with an additional unilateral VIM, and 2 unilateral VIM. The specific target varied across those patients who reported falls as primary reason of UH, where 2 had unilateral STN, 1 unilateral VIM, 1 unilateral GPi with additional unilateral VIM, and 1 bilateral GPi,

**Table 7:** Simple logistic regression analysis predicting unintended hospitalization following DBS.

Variable	Category	Total cohort	UH positive, n	Ratio	Odds ratio	P value	95% CI
Age at surgery, years	≥ 70	27	7	25.9	Reference		-
	65–69	30	5	16.7	0.623	0.384	0.08 to 1.167
	< 65	67	12	17.9	0.571	0.395	-0.09 to 1.23
Disease duration, years	≦10	41	8	19.5	Reference		-
	> 10	83	16	19.3	0.985	0.975	0.383 to 2.536
Pre-op UPDRS part III							
“Off” medication	≦ 40	63	12	19.0	Reference		-
	> 40	50	12	24.0	1.342	0.523	0.544 to 3.313
“On” medication	≦ 25	59	11	18.6	Reference		-
	> 25	56	13	23.2	1.319	0.547	0.535 to 3.252
Post-op UPDRS part III							
“Off” med “On” stim	≦ 40	54	10	18.5	Reference		-
	> 40	20	4	20.0	1.100	0.885	0.302 to 4.008
“On” med “On” stim	≦ 25	50	8	16.0	Reference		-
	> 25	44	10	22.7	1.544	0.410	0.549 to 4.341
Pre-op PDQ-39	≦ 200	39	11	28.2	Reference		-
	> 200	70	12	17.1	0.527	0.179	0.207 to 1.340
Post-op PDQ-39	≦ 200	56	7	12.5	Reference		-
	> 200	35	12	34.3	3.652	0.016	1.271 to 10.495
Change in PDQ-39	0%	63	8	12.7	Reference		-
	> 0%	27	10	37.0	4.044	0.011	1.377 to 11.874

UH: unintended hospitalization; UPDRS: Unified Parkinson’s Disease Rating Scale; PDQ-39: Parkinson’s Disease Questionnaire.

## Discussion

We hypothesized that UH and QoL would be associated with findings uncovered by an interdisciplinary DBS screening team. Twenty-one percent of patients (1 in 5) had an unintended postoperative hospitalization following DBS therapy. Other studies have revealed that regardless of DBS status, patients with PD experienced a higher rate of hospitalization when compared to age-matched controls [11–14] and that up to one-third of patients with PD will visit the emergency department or be hospitalized at least once a year [6]. Our results add to the literature on PD hospitalization, and demonstrate that patients seeking DBS therapy with major or minor concerns uncovered by interdisciplinary screening have more UH. This finding supports the potential importance of fall prevention therapy and other directed approaches for reducing morbidity in DBS patients. In fact, the rate of major and minor concerns in the UH group eclipsed 90%. Major causes of UH were variable and included depression, cognitive dysfunction, anxiety, dysphagia, balance issues, and falling. A non-significant difference in the rate of hospitalization was observed in those candidates who had DBS when compared to those who did not undergo DBS surgery, (21% vs. 19%, p = 1), however, only documented hospitalizations could be considered in this analysis.

The three most common disciplines whose concerns were associated with UH were psychology, neuropsychology and neurology. Though the other disciplines were not as vital to UH and QOL, this does not diminish the value of these services particularly to the DBS screening process. In a related study by our group looking at UH in ET DBS patients, the most common specialty of origin for concerns in UH patients were neurosurgery, physical therapy, neurology, neuropsychology, and speech. This finding underscores the importance of having a sufficiently wide array of specialties in order to address the risks associated with utilizing DBS for heterogeneous diseases often requiring different therapeutic brain targets. We suspect that much of the reasons for the importance of these disciplines hinged on the critical importance of non-motor PD symptoms which may affect up to 88% of PD patients [15]. It is difficult to reconcile that although the most common concerns raised in the UH group were mood or cognitive related, the most common cause for hospitalizations was falls.

In patients who required hospitalization following DBS, the cause was not directly DBS-related in over 80% of cases, consistent with previous findings [16]. The results of the UH group also revealed that the higher the level of concern, the higher the rate of UH. Management of the concerns uncovered by an interdisciplinary DBS team have been previously addressed in a cohort of Essential Tremor patients by our group [17], however, we would argue that PD patients with concerns raised by the screening team should undergo pre-operative counseling and closer post-operative follow-up care. These care issues were not addressed by our study. Avoidance of UH likely will include fall prevention, more frequent follow up, drug optimization, and active monitoring of pharmacological and behavioral therapy compliance [18, 19].

This study was limited by the retrospective chart review methodology which has the tendency to miss or to under-report complications, though it should be pointed out that the sample size was reasonably large, and may have somewhat protected against this bias. Additionally, the levels of concerns used in the study were determined based on the judgment of specialists who focus solely on movement disorders and thus may affect the generalizability of results to those practices that do not employ movement disorder specific specialists. However, assessments were based in part on quantitative tests. Each team evaluated the candidates based on their detailed quantitative assessment, though discussion and concerns were expressed qualitatively. Further studies should be conducted to compare qualitative and quantitative screening methodologies, though we suspect both techniques will be needed to account for the complexity of the DBS screening process. No comparisons were made of assessment outcomes between screens employing seven specialties versus those employing a more traditional approach of fewer specialties. It would be interesting to quantitate the incremental value, if any, of adding more extensive evaluations comprising more specialties. The absence of a control group (without interdisciplinary team evaluation) limited the interpretation of the results, however using a control group would not have been considered ethically reasonable. There were many different targets implanted across patients reporting falls as the main reason of UH, suggesting the possibility that other factors such as disease progression underpinned falling. A previous study reported an association between using antidepressants and the frequency of falls in PD patients; however, we did not investigate this association [20].

## Conclusion

The current study demonstrated that the DBS interdisciplinary team approach provided important information on risk for UH and improvement in QOL following DBS surgery. The success of DBS therapy is known to heavily depend on the quality of candidate selection. We would contend that detailed screenings by interdisciplinary teams may be useful for more than just patient selection. These evaluations may help to stratify risk for post-operative hospitalization and QOL outcomes.
